# Dose-Dependent Effects of Aloin on the Intestinal Bacterial Community Structure, Short Chain Fatty Acids Metabolism and Intestinal Epithelial Cell Permeability

**DOI:** 10.3389/fmicb.2019.00474

**Published:** 2019-03-26

**Authors:** Kuppan Gokulan, Pranav Kolluru, Carl E. Cerniglia, Sangeeta Khare

**Affiliations:** Division of Microbiology, National Center for Toxicological Research, United States Food and Drug Administration, Jefferson, AR, United States

**Keywords:** aloin, commensal bacterial community, antimicrobial activity, epithelial cell barrier function, short chain fatty acids

## Abstract

Aloe leaf or purified aloin products possess numerous therapeutic and pharmaceutical properties. It is widely used as ingredients in a variety of food, cosmetic and pharmaceutical products. Animal studies have shown that consumption of aloe or purified aloin cause intestinal goblet cell hyperplasia, and malignancy. Here, we tested antibacterial effects of aloin, against intestinal commensal microbiota. Minimum inhibitory concentration of aloin for several human commensal bacterial species (Gram-positive and Gram-negative) ranged from 1 to 4 mg/ml. Metabolism studies indicated that *Enterococcus faecium* was capable of degrading aloin into aloe-emodin at a slower-rate compared to *Eubacterium* spp. As a proof of concept, we incubated 3% rat fecal-slurry (an *in vitro* model to simulate human colon content) with 0.5, 1, and 2 mg/ml of aloin to test antimicrobial properties. Low aloin concentrations showed minor perturbations to intestinal bacteria, whereas high concentration increased *Lactobacillus* sp. counts. Aloin also decreased butyrate-production in fecal microbiota in a dose-dependent manner after 24 h exposure. The 16S rRNA sequence-data revealed that aloin decreases the abundance of butyrate-producing bacterial species. Transepithelial resistant result revealed that aloin alters the intestinal barrier-function at higher concentrations (500 μM). In conclusion, aloin exhibits antibacterial property for certain commensal bacteria and decreases butyrate-production in a dose -dependent manner.

HIGHLIGHTS

–Aloin exhibits antibacterial properties for certain intestinal commensal bacteria.

–In rat fecal slurry (an *in vitro* model to simulate human colon content), longer aloin exposure (24 h) decreases the butyrate production in dose dependent manner.

–The 16s rRNA sequencing data show that aloin decreased the abundance of butyrate producing bacterial species.

–Rat intestinal commensal bacteria metabolized aloin into aloe-emodin.

–Aloin altered the intestinal epithelial cells barrier integrity, however, the metabolic product of aloin - Aloe-emodin did not alter epithelial cells permeability.

## Introduction

Aloe Vera is a tropical medicinal plant that belongs to the Liliaceae family (Ding et al., 2014). Aloe is a genus that consists of 548 recognized species. Aloe leaf has been used for medicinal purposes since ancient times (Morton, 1961). The two major components of aloe leaves are the mucilaginous inner gel and the outer dermal layer. The inner gel is composed of 99% water and several bioactive molecules, vitamins, sugars, amino acids, lipids, sterols, and phenolic compounds, most of which have medicinal values (Park and Kwon, 2006). Phenolic molecules, chromones, anthraquinones glycosides, and Aloin (A and B isomer) make up approximately 30% of the outer cutaneous layer (Gutterman and Chauser-Volfson, 2000; Hamman, 2008). It is important to note that the composition of bioactive molecules in Aloe vera varies depending on the age of the plant.

In Asian and African countries, the aloe leaves or extracted juice are often used to treat infectious and inflammatory diseases (Thiruppathi et al., 2010; Akinduti et al., 2013; Megeressa et al., 2015). Aloe vera derived products are also used to treat hair loss and constipation. Studies have suggested that in certain regions of the world, aloe leaf is used to treat asthma, constipation, hypertension, and excessive perspiration (Lans, 2006). More recently, the jelly products have been incorporated as an ingredient in cosmetics, skin care products, health supplements, health beverages, herbal remedies, and also used for their wound healing, anti-inflammatory, antimicrobial, and anti-oxidant activities (Briggs, 1995). The United States Food and Drug Administration (FDA) has approved the use of aloe leaf product ingredients for natural food coloring purpose, cosmetics, and dietary polyphenolic supplements (FDA, 2008); however, the exact molecular mechanisms of its beneficial effects is poorly understood.

The intestinal epithelial layer of the gastrointestinal system operates as a physical barrier between the internal system and outside environment (Odenwald and Turner, 2013). The intestinal system also provides a microbial habitat for 10^10^ to 10^14^ commensal microbiota (Ley et al., 2006; Qin et al., 2010) per gram feces. In healthy individuals, the composition of commensal bacteria is a stable microbial community, which shapes the development of the local immune system in the gut. In addition, the microbiome aids short chain fatty acid (SCFA) production by degrading undigested polysaccharides and other food materials. Bacterial derived butyrate serves as a major energy source for intestinal epithelial cell growth and maintenance of barrier function (Zheng et al., 2017). Progressions of intestinal disorders and diseases have been associated with shifts in the normal intestinal microbiome and dysbiosis in butyrate-producing intestinal bacteria (Clemente et al., 2012; Zheng et al., 2017). The metabolites of the ingested aloin can be absorbed by intestinal epithelial cells (Park et al., 2009) while traveling through the gastrointestinal tract (GIT) and these molecules may interact with commensal microbiota and alter intestinal homeostasis.

In the GIT, ingested aloin can be metabolized into deglycosylated aloe-emodin by intestinal bacteria (Che et al., 1991). The purified phenolic aloe products and its metabolites have free hydroxyl methyl groups which may interact with other hydroxyl groups or glucuronide enzymes to form glucuronide conjugates. Glucuronate derivatives are more stable than sulfate derivatives and require more time to clear from the system. *In vitro* permeability studies have revealed that intestinal epithelial cells absorb aloin and its metabolites in the following order: alosin > aloe-emodin > aloin (Park et al., 2009). These metabolites retain the phenolic structures that are considered bioactive molecules. Studies have also demonstrated that aloin exhibits antimicrobial and antimalarial properties against many pathogenic organisms (Asamenew et al., 2011; Abeje et al., 2014; Oumer et al., 2014).

Few studies provide evidence that Aloe vera products are safe to use and do not cause toxic effects (Sehgal et al., 2013). In contrast, many other studies have shown that the ingestion of aloe-products causes intestinal abnormalities (Boudreau et al., 2013, 2017; Sehgal et al., 2013). Since aloin and its metabolites exhibit antimicrobial properties, it is reasonable to conduct safety assessment of aloe-derived products for intestinal microbes, as well. Previous rodent studies have provided evidence that administration of aloe leaf or purified polyphenolic compounds causes lesions, cytotoxicity, and the progression of adenocarcinoma in the intestine (Xia et al., 2007; Boudreau et al., 2017). Interestingly, these intestinal abnormalities were found to be more predominant in males than females (Xia et al., 2007). Thus, systematic approaches are necessary to determine the concentration of aloin that can cause detrimental effects on intestinal microbiota. Specifically, the concentration of aloin responsible for the alteration of the intestinal microbial community and the integrity of the gastrointestinal system is not known. Additionally, there is a knowledge-gap regarding which experimental approaches should be used to assess whether the barrier function of the intestinal epithelial layer is compromised due to xenobiotic exposure and/or its metabolites. Furthermore, the antibacterial effect of aloin on intestinal commensal bacteria and production of SCFAs from undigested fiber food materials also remains to be answered.

In the present study, we hypothesized that aloin and its metabolites may cause detrimental effects to the gastrointestinal system. To test our hypothesis, we have employed *in vitro* experimental approaches to evaluate aloin toxicity toward commensal bacteria and intestinal epithelial cells. In this study, our main objectives were fivefold: (i) to evaluate if there are any differences in the antibacterial properties of aloin between aerobic, facultative, and anaerobic human intestinal bacteria that are predominant in the intestine, (ii) to examine the effect of aloin on changes in the aerobic and anaerobic bacterial community structures using an *in vitro* rat fecal culture model and 16s rRNA sequencing, (iii) to examine the effect of aloin on SCFA production by rat intestinal bacterial community, (iv) to evaluate the effect aloin on intestinal permeability by *in vitro* methodology, and (v) to analyze the metabolism of aloin by intestinal commensal bacteria.

## Materials and Methods

### Antibacterial Activity of Aloin on Pathogenic Bacteria and Human Intestinal Bacteria Growth

To test the antimicrobial properties of aloin, we used a reference *Escherichia coli* strain as a test organism. Overnight grown *E. coli* J53 strain was sub-cultured in tryptic soya agar (TSA) media to reach mid log phase, then diluted and plated (1 × 10^5^ cells/well) into 96 well culture plate. Aloin stock (CAS 1415-73-2 procured from Pure Chemistry Scientific Inc., Houston, TX, United States) solution was prepared (10 mg/ml) by adding ethanol and culture media in 1:4 ratio; then a twofold dilution method was used to obtain 2mg/ml to 0.015 μg/ml concentration in culture plates. Control wells received only media and ethanol (equivalent concentration of ethanol present in 2 mg/ml aloin solution). The negative control was media alone without bacteria and served as a correction value for background reading. Control wells containing 2 mg/ml aloin and media were also included to detect background absorption of aloin. Plates were placed in BioTek plate reader (Cytation, BioTek Instruments, Inc., Winooski, VT, United States) and OD was recorded at 600 nm with 5 min intervals for a 24 h period to evaluate growth.

We selected six commensal bacterial strains from five major bacterial phyla based on its functionality in the intestine. These human commensal bacteria were obtained from ATCC (ATCC, Manassas, VA, United States) and included *Bifidobacterium longum* (*ATCC*^®^ *15707*^TM^), *Lactobacillus acidophilus* (*strain ATCC 700396/NCK56/N2/NCFM*), *Enterococcus faecium* (*ATCC*^®^ *19434*^TM^), *Bacteroides thetaiotaomicron* (*ATCC*^®^ *29148*^TM^), *Akkermansia muciniphila* (*ATCC*^®^ *BAA-835*^TM^), and *Eubacterium* sp. (*ATCC*^®^ *BAA-148*^TM^). These strains were cultured in specific bacterial growth media as recommended by ATCC. The anaerobic bacteria were cultured in the anaerobic chamber. The facultative bacteria were cultured in partially anaerobic condition at 37°C.

### Evaluation of Minimum Inhibitory Concentration (MIC) of Aloin Against Intestinal Commensal Bacteria

Bacterial strains were inoculated overnight in the respective culture media at 37°C in a shaker as recommended by ATCC. Periodic OD_600_ measurements were taken to evaluate bacterial growth and the incubation was halted when the organism growth reached mid-log phase. Bacterial cultures were diluted using respective culture media and adjusted to a density of approximately 1 × 10^5^ cells/ml, after which, 100 μl (10,000 cells) were plated in each well of the 96 well plates. Aloin was prepared with 1:4 ratios of ethanol and respective culture media and added twofold dilutions starting from 4 mg/ml (9.56 mM) to 3.98 μg/ml (0.019 mM). Positive (bacterial culture without aloin) and negative controls (media alone and aloin alone without bacterial culture) were included in each experiment. Plates were placed in BioTek plate reader and OD was recorded at 600 nm with 5 min intervals for 24 h period to evaluate MIC. The MIC value (i.e., when aloin concentration totally inhibited bacterial growth) of aloin against intestinal commensal bacteria was determined.

### Visualization of *L. acidophilus* Viability Using Acridine Orange and Ethidium Bromide Fluorescence

After the assessment of bacterial growth, the wells containing *L. acidophilus* were stained for detection of cell death according to the protocol developed by Ribble et al. (2005). Acridine orange (2 μg/ml) and ethidium bromide (2 μg/ml) dyes were added to each well and incubated at 37°C for 15 min. The stained cells were visualized and photographed using an inverted fluorescence microscope (EVOS, Life Technologies, San Diego, CA, United States) to differentiate between live (green) and dead (red) bacteria.

### *In vitro* Microgravity Rotary Culture Conditions of Fecal Microbiota

Fecal samples were collected post-mortem from the colon of three male Sprague Dawley rats (aged 4–6 months) who undergo regular surveillance at NCTR. As these fecal samples were collected post-mortem from the animals, an approval from the Institutional Animal Ethics Committee was not required.

To mimic the intestinal continuous movements, we chose a rotary cell culture system (RCCS) (Synthecon Inc., Houston, TX, United States) for the interaction of aloin with intestinal bacteria. The RCCS was developed for zero gravity (space) research by Wolf et al. (1988). This system was specifically designed to grow long term culture of functional primary human liver cells under low microgravity environment with low shear force, high mass transfer and 3-D cell culture of dissimilar cell types. In the present study, we used the same technology, however, customized the vessel to mimic an enclosed system with an anaerobic environment. The RCCS was placed inside of anaerobic chamber with low speed to mimic peristaltic movement of the intestine. We employed this system to study the effect of aloin on intestinal bacteria to mimic anaerobic environment of colon. We prepared aloin at 10 mg/ml concentration as described in “Materials and Methods” Section “Antibacterial Activity of Aloin on Pathogenic Bacteria and Human Intestinal Bacteria Growth.” Fecal slurry (3%) was prepared using low carbohydrate medium (LCM) under anaerobic conditions as described earlier (Kim et al., 2011; Jung et al., 2018). The fecal slurry was aliquoted into four equal volumes. The final concentrations of all four fecal slurries were adjusted to 3% using LCM. The fecal slurry was transferred into 10 ml custom made sterile anaerobic disposable culture vessels (four vessels) using a syringe. Care was taken to remove air bubbles. These vessels were fixed into a RCCS and the rotation speed was adjusted to 7 rpm/min. The experiment preparations and setup were conducted in an anaerobic chamber. The control sample received 400 μl of ethanol, which is equivalent to the highest concentration of ethanol that is present in 2 mg/ml aloin solution. In the remaining three vessels, aloin was added (0.5, 1, and 2 mg/ml, respectively) to analyze changes in the intestinal bacterial community structure, the concentrations were based on the MIC obtained from *E. coli* bacterial growth curve. A portion of the samples from these vessels were collected at 3, 6, and 24 h time points to analyze changes in the bacterial structural community for both aerobic and anaerobic bacteria, SCFAs production, and aloin metabolism by intestinal bacteria.

### Bacterial Cultures From Rat Fecal Slurry Treated With Aloin

Fecal samples were collected from all rotary vessels as indicated in Section “*In vitro* Microgravity Rotary Culture Conditions of Fecal Microbiota.” To analyze the bacterial composition, 100 μl from each sample was serially (10-fold) diluted in dilution blank solution. The total aerobic and anaerobic bacterial populations were quantified using selective culture media plates (TSA and Brucella Blood Agar, respectively). These samples were also plated in a selective bacterial culture medium for anaerobic [BBE (for *Bacteroides*) and Bifido (for *Bifidobacterium* genus)], and facultative bacteria (LRMS). Culture plates were incubated at 37°C. The aerobic and facultative bacterial plates were removed after 24 h. The anaerobic bacterial plates (BBE and Bifido) were removed after 72 h from the anaerobic chamber. Bacteria were then enumerated using a colony counter. The bacterial count obtained from control group was compared to that of the three experimental groups at each time point.

### Sample Preparation to Measure Short Chain Fatty Acids

The HPLC equipment, 1260 DAD LC Agilent (Agilent Technologies, Santa Clara, CA, United States), was used to measure SCFAs. Standards of succinic acid, lactic acid, acetic acid, propionic acid, isobutyric acid butyric acid, valeric acid, isovaleric acid, and hexanoic acid were obtained from Sigma-Aldrich (St. Louis, MO, United States). Standards were prepared freshly at 1 M concentration and 20 mM spiked in control fecal supernatant. Before analyzing the test samples, retention times for standards were calibrated using Aminex HPX-87H column (300 × 7.88 mm) (Bio-Rad, Richmond, CA, United States). The column was maintained at 65°C and the column flow rate (0.6 ml/min) and molecules were monitored continuously at 210 nm. The mobile phase was composed of an isocratic H_2_SO_4_ solution (2.5 mM) for 50 min. An aliquot (1 ml) of fecal slurry was removed at 3, 6, and 24 h for SCFA analysis by HPLC method (Gordon et al., 1982). Samples were immediately centrifuged at 15,000 rpm for 10 min at 4°C and supernatants were collected and filtered through a 0.45 μm membrane-filter. A 20 μl of sample solution was injected into HPLC column to analyze SCFAs metabolites.

### Sample Preparation for Assaying Aloin Metabolism by Commensal Bacteria

After evaluating the MIC value from pure bacterial culture, samples were centrifuged to remove the bacterial pellet. The supernatant was transferred in a new tube then kept in speedvac to remove culture media. Aloin and its metabolites were extracted using methanol. Aloin and aloe-emodin standards were prepared freshly and evaluated for the retention time using Zorbox-SB C18 HPLC column. These standards were also spiked in control bacterial culture media, retention time for extracted aloin and aloe-emodin was analyzed as stated earlier. Experimental samples were analyzed by HPLC column to evaluate aloin and its metabolites. Column temperature was kept at 40°C. Buffer A was 95% water and 5% Acetonitrile containing 0.1% TFA. Buffer B was 95% Acetonitrile and 5% water containing 0.1% TFA. Flow rate was 0.2 ml/min and absorbance was monitored at 260 nm.

### Extraction of DNA From Fecal Samples

Fecal DNA was extracted following the protocol described earlier with slight modifications (Khare et al., 2004; Williams et al., 2015). One ml of fecal sample was collected from each experimental group at 3, 6, and 24 h. DNA was extracted from fecal samples as described earlier. The pellet was suspended in the DNase and RNase free water and DNA was quantified using a Nanodrop ND-1000 spectrophotometer (Nanodrop, Wilmington, DE, United States). In addition, dsDNA was quantified by Qubit (Thermo Fisher Scientific, Waltham, MA, United States). This highly purified DNA was used as template for 16s sequencing for microbial population.

### 16S rRNA Gene Sequencing of Bacterial Population

Highly purified fecal DNA was used to analyze change in bacterial community due to aloin exposure. The V4-variable region of 16S rRNA gene was amplified using PCR primers 515/806. Pooled and purified PCR products were used to prepare Illumina DNA library. The output of the DNA sequence reads were joined and subsequently, the barcodes were depleted and then sequences <150 bp were removed. Sequences with ambiguous base calls were also removed for data analysis. Sequences were denoized, operational taxonomic units (OTUs) generated and chimeras were removed. OTUs were defined by clustering at 3% divergence (97% similarity). Final OTUs were taxonomically classified using BLASTn against a curated database derived from RDPII and NCBI.

### Intestinal Epithelial Cell Culture

T84 cells (ATCC^®^ CCL-248^TM^), a human colorectal carcinoma cell line, were obtained from ATCC. Cells were cultured in complete growth media reported previously (Gokulan et al., 2016, 2017), which was composed of Dulbecco’s Modified Eagle Medium (DMEM)/F-12 medium supplemented with L-glutamine and HEPES (ATCC), with added 5% fetal bovine serum, penicillin/streptomycin, and Fungizone. Initially, a 75 cm^2^ cell culture flask was used to grow the cells until they reached 70–80% confluency. Cells were detached with 0.25% trypsin-EDTA solution and washed twice with DMEM/F-12 medium. The media was decanted, and cell pellet was suspended with culture media. Cells were counted, then seeded (2.0 × 10^5^ cells/well) into transwells and maintained in a 37°C incubator with 5% CO_2_ and 95% humidity until transepithelial electrical resistance (TER) values were stabilized.

### Transepithelial Electrical Resistance (TER)

The TER measurement was performed as reported previously (Adams et al., 1993; Youakim and Ahdieh, 1999; Bruewer et al., 2003; Donato et al., 2011; Khare et al., 2012; Williams et al., 2016). Briefly, T84 cells were seeded in the apical compartment of 6.5 mm, PFTE, collagen-coated transwells inserts (Corning, Corning, NY, United States) at a concentration of 2 × 10^5^cells/well. Complete cell culture media was added to apical (0.2 ml) and basal reservoirs (0.8 ml) and cells were allowed to grow for 5–7 days to polarize (Williams et al., 2016). Then antibiotic-free medium was added to apical and basal reservoirs and TER was measured periodically using a STX electrode probe and EVOM2 Epithelial Voltohmmeter (World Precision Instruments, Sarasota, FL, United States). Once the wells had reached approximately 800–1000 Ω/cm^2^ the medium was changed and cells were allowed to equilibrate for 3 h. Aloin was dissolved in ethanol and media in a 1:4 ratio (10 mg Aloin dissolved in 0.2 ml ethanol and 0.8 ml of culture media at pH 7.2 or citrate buffer pH 4.9) and further diluted in cell culture media for the TER studies. The control wells contained 0.8% of ethanol (500 μM Aloin contains 0.8% ethanol). Baseline TER reading was taken before adding test compound as an initial value, then with the following aloin concentrations (0.05, 0.5, 1.5, 5, 50, and 500 μM) in apical compartments. TER measurements of individual transwells were taken before and after exposure to aloin in all wells (including controls) at 1, 2, 3, 4, 24, and 48 h post-exposure.

### Statistical Analysis

The bacterial growth curve data was analyzed using one-way ANOVA to determine statistical significance in culture treated with different concentration of aloin. One-way ANOVA was also used to find out statistical significance on SCFAs production.

During 16S rRNA analysis, data were normalized by median and auto-scaling (mean centered/standard deviation of each variable). The effect of aloin-exposure on the bacteria representative of specific-phylum, genus and species level was calculated as the percent abundance. Next, a one-way ANOVA was performed to obtain significant differences between the experimental groups and control for Phyla, genes, and species level. For Genus and Species level analysis, Orthogonal Partial Least Squares Discriminant Analysis (PLS-DA) was used to observe the separation between different experimental groups and to assess the similarities within an experimental group, as described earlier (Al-Momani et al., 2016; Rabbi et al., 2016; Gokulan et al., 2018) The beta diversity was provided as the intensity of heatmap, where the clustering result shown as heatmap (distance measure using euclidean, and clustering algorithm using ward’s method). The heatmap here is expressed by the abundance of genus or species while comparing the controls and treatment groups (individual columns depict the average of one experimental group).

## Results

### Antimicrobial Activity of Aloin Against *E. coli* J53 Strains

**FIGURE 1:**
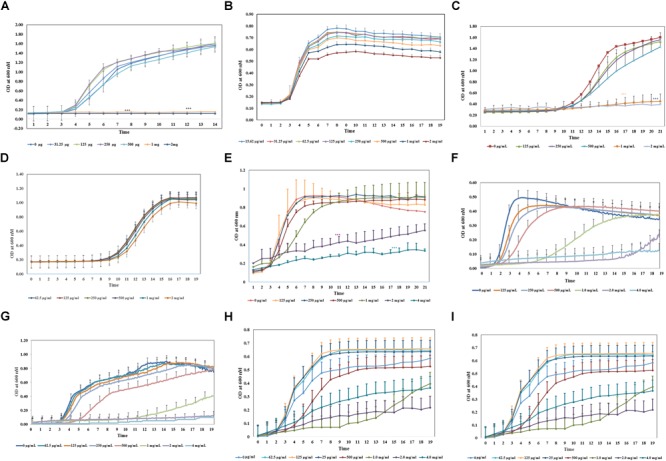
Antimicrobial property of aloin on *in vitro* cultured bacterial species. All bacterial cultures were incubated with two-fold serial dilution of aloin starting a concentration of 2 mg/ml to 0.03 μg/ml **(A–D)** or 4g/ml to 0.015 μg/ml **(E–I)**. The data presented here were average of five independent experiments. Values of *p* < 0.05 or below indicated as a statistical significance compared to control group or among the experimental groups. Asterisks indicates statistical significance (^∗^*p* < 0.05, ^∗∗^*p* < 0.01, ^∗∗∗^*p* < 0.001). **(A)** *E. coli* J53 was grown under aerobic conditions. **(B,C)** *L. acidophilus* was grown under aerobic conditions or anaerobic conditions, respectively. Aloin was unable to kill *L. acidophilus* under anaerobic conditions. **(D)** *B. longum* was grown under anaerobic conditions. The figure shows that only higher concentration of aloin was able to decrease *Bifidobacterium* growth under anaerobic conditions. **(E,F)** *E. faecium* was grown under aerobic or anaerobic conditions, respectively. The figure shows that aloin was able to decrease *E. faecium* growth under aerobic conditions even at 4 mg/ml concentration but unable to prevent bacterial growth, however, aloin was able to kill *E. faecium* growth under anaerobic conditions. **(G)** *B. thetaiotaomicron* was grown under anaerobic conditions. The figure shows that aloin was able to kill *B. thetaiotaomicron* growth under anaerobic condition and the MIC value was 1 mg/ml. **(H)** *A. muciniphila* was grown under anaerobic conditions. The figure shows that aloin decreases *A. muciniphila* growth under anaerobic conditions. **(I)** *Eubacteria* sp. was grown under anaerobic conditions. The figure shows that aloin decreases *Eubacterium* sp. growth under anaerobic conditions.

To assess the antimicrobial activity of aloin against the reference strain *E. coli* J53, a sub-culture was incubated with aloin from 2 mg/ml to 0.015 μg/ml by twofold serial dilution. The bacterial growth was monitored for 24 h with data collection at 5 min intervals. The growth curve kinetic data of control *E. coli* at low aloin concentrations indicated that there was an initial 3 h lag phase, an exponential phase for 5–6 h, and stationary phase at 14 h. At the two higher concentrations of aloin (1 and 2 mg/ml), treated wells showed the complete inhibition of *E. coli* growth, which was statistically significant (*p* < 0.001) as compared to bacteria exposed to lower concentrations (0.015 μg/ml to 0.5 mg/ml) of aloin (Figure 1A). Wells containing only 2 mg/ml of aloin were measured and had absorbance values between 0.1 and 0.15, which was subtracted from experimental wells treated with 2 mg/ml of aloin. The aloin MIC value for *E. coli* strain was 1 mg/ml.

### Minimum Inhibitory Concentration of Aloin Against Intestinal Commensal Bacterial Strains

The same experimental approach was carried out to determine the minimal inhibitory concentration of aloin for human commensal bacteria (i.e., *L. acidophilus, B. longum*; *E. faecium, B. thetaiotaomicron, A. muciniphila*, and *Eubacterium* sp.). For some bacteria, aloin concentration was increased above 2 mg/ml to 4 or 8 mg/ml since several bacterial species were not susceptible to 1 or 2 mg/ml of aloin. These experiments were conducted aerobically, anaerobically, or microaerophilic based on the growth condition requirements of the bacteria.

*Bifidobacterium longum* growth curve revealed that 1 and 2 mg/ml of aloin exposure slightly reduced bacterial growth, whereas lower concentrations of aloin had no effect on bacterial growth (Figure 1B). *B. longum* exposed to 4 mg/ml aloin also showed growth pattern similar to 2 mg/ml aloin exposure. Overall, the difference in the growth of *B. longum* was not statistically significant. This result showed that the aloin showed poor antibacterial activity toward *B. longum*; hence, no MIC value was recorded in the bacterial growth curve. The growth kinetic results suggest that under aerobic conditions, 1 and 2 mg/ml concentrations of aloin efficiently inhibited *L. acidophilus* growth (Figure 1C). The antimicrobial effect of these two concentrations were statistically significant (*p* < 0.05) compared to lower concentrations (0.015 μg/ml to 0.5 mg/ml). The growth curve revealed an initial 11 h lag phase for samples exposed to all concentrations of aloin. *L. acidophilus* grew faster after the lag phase and its exponential growth phase was 3–4 h, followed by a stationary phase. Under anaerobic conditions, the lag and log phase times were similar for aerobic incubations; however, under anaerobic conditions, aloin did not have a similar antimicrobial effect on *L. acidophilus* (Figure 1D). Wells treated with higher concentrations of aloin (above 2 mg/ml) also had a similar growth curve to that observed in wells treated with lower concentrations. These data clearly suggest differential antibacterial properties of aloin under aerobic versus anaerobic culture conditions. *L. acidophilus* grown aerobically had an MIC value of 1 mg/ml.

In contrast, the result of aerobic growth data of *E. faecium* in the presence of aloin was significantly different than *L. acidophilus.* Specifically, the log phase of *E. faecium* varied between the lower and higher concentrations of aloin (Figure 1E). At lower concentrations (125–250 μg/ml) the lag phase was approximately 2 h and the stationary phase was within the 6 h time point. During exposure at higher concentrations (500 μg/ml to 4 mg/ml), the exponential growth starting time was delayed by 3–4 h and bacterial growth remained in the exponential phase until 20 h with a slower growth rate. *E. faecium* exposed to 1, 2, and 4 mg/ml had a statistical significant difference (*p* < 0.001) in a dose-dependent manner compared to groups treated with low concentrations of aloin. However, in 1 mg/ml treated samples, statistically significance was observed until 7 h, due to increased bacterial growth, but no significance difference was observed after the 7 h time point. Cultures treated with 2 and 4 mg/ml of aloin had lag phase for 8 h, after which, bacterial growth started slowly; hence, no MIC value was recorded for *E. faecium* under aerobic conditions. Under anaerobic conditions, the growth curve data revealed that lag phase was dose dependent. At low concentration (0.125 mg/ml), lag phase was 2 h, but at a higher concentration (1 mg/ml) the lag phase was 7 h (Figure 1F). The higher concentration (2 mg/ml) of aloin completely inhibited the bacterial growth. In 1 mg/ml exposed wells, the log phase was delayed more than 5–9 h and reached the stationary phase after 14 h. The MIC value of aloin for *E. faecium* fell between 2 and 4 mg/ml concentrations.

We also examined the effect of aloin on *Bacteriodes* growth. The growth curve data indicates that MIC concentration of aloin for *Bacteriodes* was 2 mg/ml; this concentration completely inhibited the bacterial growth and was statistically significant (*p* < 0.001) when compared to the effects of lower aloin concentrations (Figure 1G). In Bacteroides cultures that were exposed to 1 mg/ml of aloin, the lag phase was approximately 11 h, then the log phase began and continued for 24 h. For the 500 μg/ml treated cultures, the lag phase was 5 h, then log phase continued for 17 h and was statistically significantly different from the effects of lower aloin concentrations. After 18 h, it reached stationary phase and growth was similar to that of the other lower aloin concentrations.

*Akkermansia muciniphila* is a Gram-negative anaerobic bacterium that belongs to phylum *Verrucomicrobia*, which colonizes in the intestine and accounts for approximately 1–5% of commensal bacteria in the colon. In healthy individuals, its main function is degrading the mucin and producing several metabolic products (Derrien et al., 2008). We tested the antimicrobial effect of aloin on *A. muciniphila*. The bacterial growth curve showed a dose-dependent growth inhibition. The growth curve data also revealed that the lag phase was dose-dependent and the highest two concentrations were statistically different (*p* < 0.05) compared to lower concentrations (Figure 1H). The MIC value of aloin for *A. muciniphila* was 2 mg/ml and the bacterial growth was completely inhibited.

**FIGURE 2:**
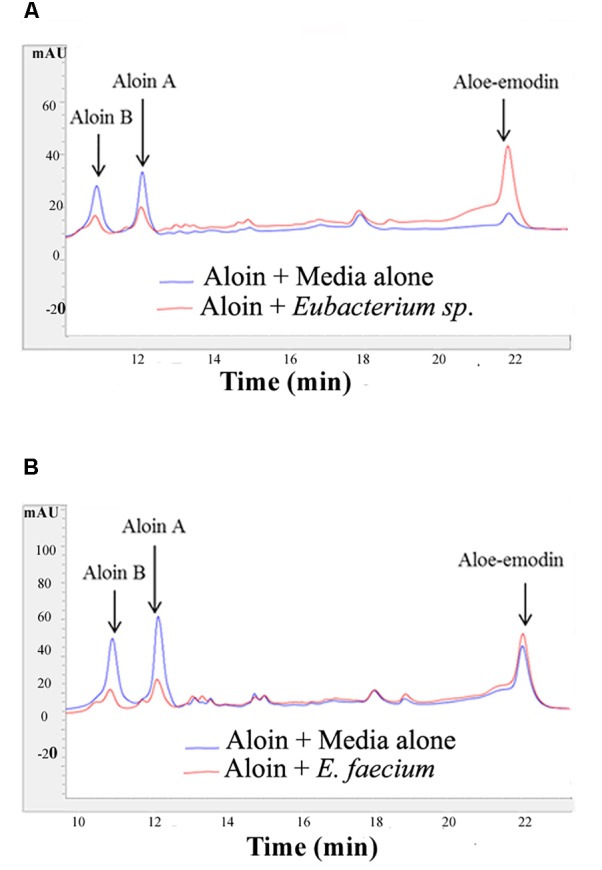
Aloin metabolism by intestinal bacteria *Eubacterium* sp. and *E. faecium* after incubation. **(A)** Top panel shows aloin metabolism by *Eubacterium* sp. under anaerobic conditions. **(B)** The bottom panel shows aloin metabolism by *E. faecium* under anaerobic conditions. The arrow shows the peak area and retention times of aloin isoforms and aloe-emodin.

The growth curve data of *Eubacterium* sp. revealed that during the initial 5 or 6 h time period, bacterial growth rate was very slow in wells incubated with 2 and 4 mg/ml aloin concentrations, which was statistically significant (*p* < 0.05) compared to those in wells treated with lower aloin concentrations. *Eubacterium* spp. ability to metabolize aloin slowly (after 5 h) could result in accumulation of glucose, which bacteria use as a nutrient for growth and multiplication. The bacterial growth of 4 and 2 mg/ml aloin treated wells was higher than in lower aloin concentrations after 13–14 h exposure and continued the log phase up to 20 h (Figure 1I). The MIC values were unable to be determined likely because aloin was metabolized as noted previously (Che et al., 1991; Pogribna et al., 2008). We confirmed this observation by analyzing metabolism of aloin in *Eubacterium* cultures by HPLC (Lobbens et al., 2016) (Figure 2A). The HPLC analysis revealed disappearance of aloin-A and aloin-B (retention time was 10.30 and 8.50 min, respectively) and appearance of aloe-emodin (retention time was 22.1 min), which provides evidence that *Eubacterium* sp. metabolized aloin (Figure 2B).

### Effect of Aloin on Total Aerobic Bacterial Population in Rat Fecal Samples

**FIGURE 3:**
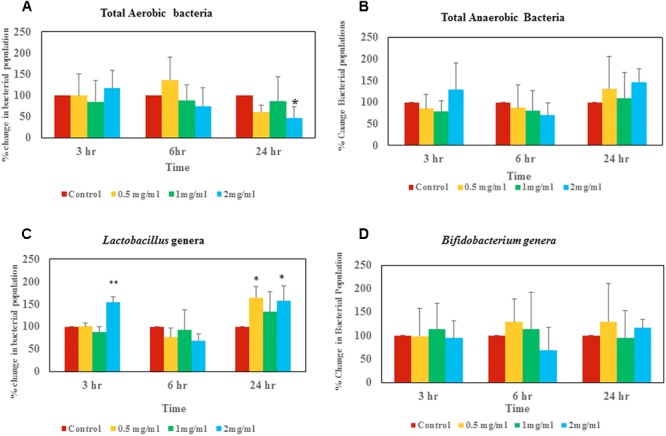
Changes of live bacterial counts after aloin exposure in rat fecal samples. The data show % change in live bacterial population compare to control. **(A)** Aerobic live bacterial counts after aloin treatment, **(B)** anaerobic live bacterial counts after aloin exposure, **(C)** live *Lactobacillus* genera counts under microaerophilic environment after aloin exposure, and **(D)** live bacterial counts of *Bifidobacterium* genera after aloin exposure. The data shown here are average of four individual experiments. Values of *p* < 0.05 or below indicated as a statistical significance. Asterisks indicates statistical significance (^∗^*p* < 0.05, ^∗∗^*p* < 0.01).

Bacterial counts revealed that at the 3 h time point there was not a significant difference in total aerobic bacterial counts between the control group and samples treated with aloin (Figure 3A). Live total aerobic bacterial count obtained from rat fecal slurry incubations was similar to human commensal pure culture aerobic bacterial growth curve data, in which, most of the aerobic bacterial growth showed initial 3 h lag phase except *E. faecium*. In *E. faecium*, the growth curve showed that lag phase was 1.5 h. This can be correlated to differential effect of aloin on commensal bacteria, as we observed in the growth curve. Samples collected at the 6 h time point showed a slight increase in bacterial populations in control samples and samples incubated with 0.5 mg aloin. In contrast, aerobic bacterial count decreased in the 1 and 2 mg/ml aloin treated samples, but was statistically insignificant. This result further supports the *in vitro* bacterial growth curve results. For example, the *B. longum* growth curve indicated that aloin (2 mg/ml) decreased the bacterial growth by 10% and in *E. faecium* growth was inhibited at a higher concentration. The samples collected at 24 h had similar bacterial counts as that of 6 h control groups. In contrast, the bacterial count of aloin treated samples decreased and the effect was significant (*p* < 0.05) in the 2 mg/ml treated group as compared to the 0.5 and 1 mg/ml treated samples (Figure 3A). Overall, the bacterial count revealed that 2 mg/ml aloin treated samples experienced a time and dose dependent growth inhibition of aerobic bacteria. This observation was consistent with *in vitro* bacterial growth culture results, which suggested higher concentrations had antimicrobial property.

### Effect of Aloin on Total Anaerobic Bacterial Populations in Rat Fecal Samples

Anaerobic bacterial count data suggests that at the 3 h time point, 0.5 and 1 mg/ml aloin treated samples had low bacterial counts compared to control groups (Figure 3B). In contrast, 2 mg/ml treated samples had higher bacterial counts than other groups (*p* < 0.05).

### Effect of Aloin on *Lactobacillus* in Rat Fecal Samples

At the 3 h time point, samples treated with 2 mg/ml aloin had slightly higher bacterial counts compared to other groups, which was statically significant (*p* < 0.01) (Figure 3C). In contrast, at the 24 h time point live bacterial counts indicated that all experimental groups had increased bacterial population; however, only 0.5 and 2 mg/ml treated samples were statistically significant.

### Effect of Aloin on *Bifidobacterium* in Rat Fecal Samples

*Bifidobacterium* bacterial culture dosed with aloin revealed that there was not a significant change in bacterial counts in samples that were collected at 3 h. Samples that were collected at the 6 h time point had slight increase in bacterial count in the 0.5 and 1 mg/ml aloin treatments. Samples treated with 2 mg/ml aloin had less bacterial counts, but were statistically insignificant (Figure 3D). Samples that were collected at the 24 h time point had higher bacterial counts compared to control groups, but were found to be statistically insignificant due to individual variation.

### The Effect of Aloin on the Production of Short Chain Fatty Acids

**FIGURE 4:**
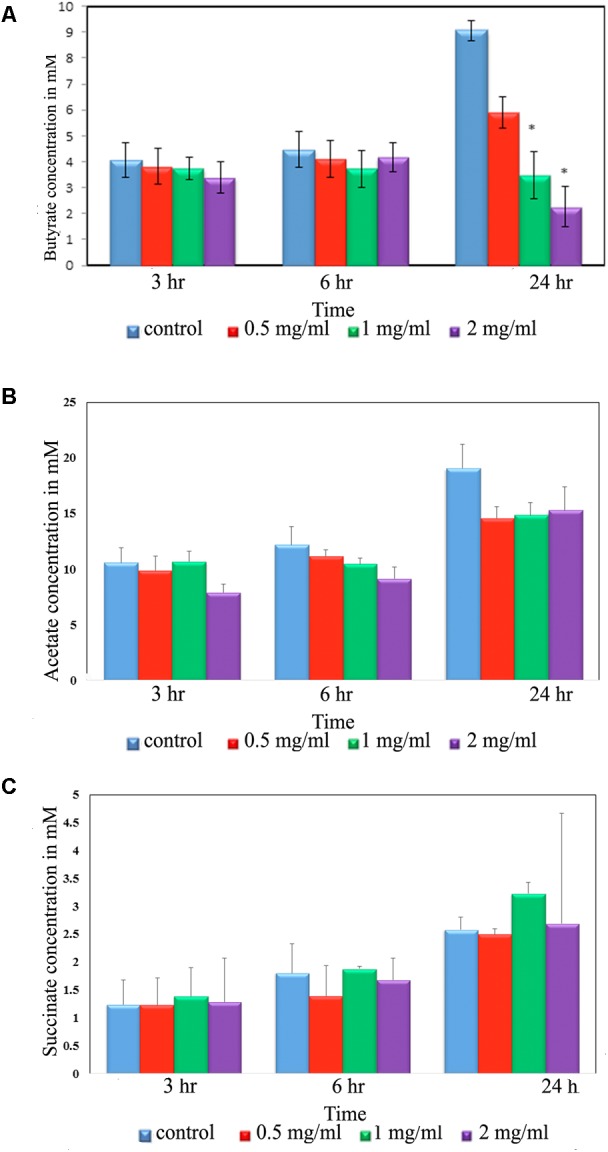
Short chain fatty acid (SCFA) production by rat intestinal bacteria after exposure to aloin. Fecal samples for SCFA analysis were collected at three different time points (3, 6, and 24 h). The SCFA production was measured by HPLC method. The presented data are averages of four independent experiments. **(A)** Butyrate production, **(B)** acetic acid production, and **(C)** succinate production. Values of *P* < 0.05 or below considered as statistically significant. Asterisks indicates statistical significance (^∗^*p* < 0.05).

Intestinal bacteria metabolize the undigested food materials and produce SCFAs (Wong et al., 2006). Hence, we analyzed acetic, butyric, succinic, lactic, propionic, isobutyric, valeric, hexnoic, and isovaleric acid SCFAs production from the fecal slurry treated with aloin. Importantly, butyrate has been known to play a central role in the homeostasis of intestinal epithelial cells (Kelly et al., 2015). Our results suggest that butyrate production decreased due to aloin exposure (Figure 4A); however, we could not observe any statistical significance for the other SCFAs [acetic acid and succinic acid (Figure 4B,C)]. The result revealed that at the 3 and 6 h time points, there was no change in the butyrate production either in the control or in experimental groups. In contrast, the 24 h samples revealed a decrease in butyrate production in a dose dependent manner. Specifically, 1 and 2 mg/ml aloin treated samples showed statistical significant as compared to 0.5 mg aloin treated samples (Figure 4A).

### The Effect of Aloin on Bacterial Phyla as Assessed by 16s rRNA Sequencing

**FIGURE 5:**
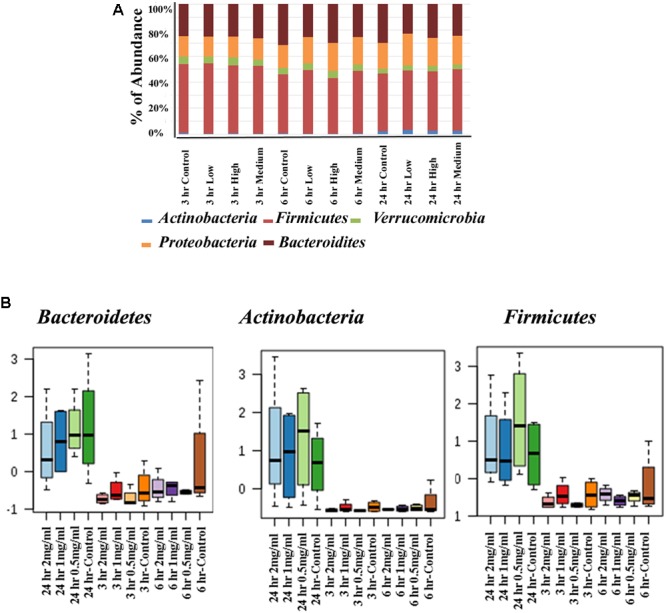
Changes in intestinal bacterial community structure due to aloin treatments. **(A)** Top panel stack bar diagram shows the abundance of bacteria at phyla level due to aloin treatment. Specifically, *Actinobacteria* at 24 h in aloin treated samples (shown in blue) over control group. **(B)** The bottom panel box diagrams show the comparative abundance of three bacterial phyla at 24 h of aloin treated cultures.

The human intestinal bacterial growth curve data revealed that antibacterial property of aloin varies between aerobic and anaerobic conditions for the same bacterial species (for example *Lactobacillus* and *Enterococcus*). For global analysis of the bacterial populations, 16s rRNA sequencing was conducted to evaluate antimicrobial effect of aloin in rat intestinal commensal bacteria. The phyla level analysis revealed that *Firmicutes, Bacteroidetes*, and *Proteobacteria* were the major phyla that contributed more than 95% of the bacterial population in all samples (Figure 5A upper panel). In addition, the analysis also revealed the contribution of *Verrucomicrobia* and *Actinobacteria* to a small percentage. Evaluation of abundance changes at the phyla level for controls and aloin treated samples suggested variations between treatment groups and exposure times. Some major differences in *Firmicutes, Bacteroidetes*, and *Actinobacteria* phyla were observed in aloin treated samples (Figure 5 lower panel). Specifically, the abundance of these three bacterial phyla was significant changes in samples collected at the 24 h time point for all three aloin concentrations (0.5, 1, and 2 mg/ml). The abundance of phylum *Firmicutes* decreased as the aloin concentration increased with time (6 and 24 h). An interesting observation was that the abundance of *Actinobacteria* was more prominent at 24 h time point than at 3 and 6 h time points. This phylum was found more abundant in aloin treated samples than the control (see Figure 5A upper panel).

### The Effect of Aloin on Bacterial Genus Level as Assessed by 16s rRNA Sequencing

**FIGURE 6:**
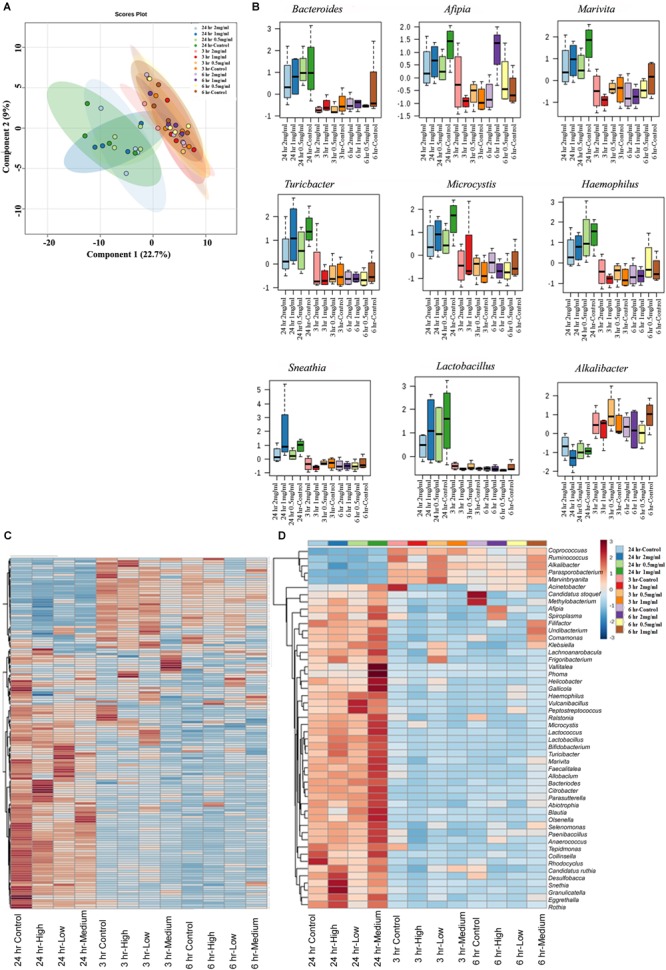
The effect of aloin on intestinal bacterial community at genus level. **(A)** The PCA plot shows clear segregation of bacterial community at the genera level at 24 h time point samples not observed in the samples from other two-time points. Samples collected from both 3 and 6 h time points are grouped tightly together. This analysis call for the *n* = 4. Here the *X*-axis is the bacterial genera and the *Y* variables are observations of different treatment groups compared together. **(B)** These figures show that aloin exposure alters the bacterial community either by increased or decreased bacterial abundance at the genus level. Here we show representative examples for high and low abundance bacterial genera due to aloin treatment. **(C,D)** The displayed heat map provides an in-built visualization of the OTU abundance and displays the similarities and differences in data subsets. In the heat map, each colored cell represents abundance of bacterial community in each experimental groups/samples (beta-diversity). **(C)** The left panel of heat map shows the abundance of bacterial population at the genus level in control and aloin treated experimental groups. **(D)** The right panel (heat map) shows the top 50 bacterial genera that altered (either increased or decreased) due to aloin treatment.

Next, we assessed similarity/differences among aloin treated experimental groups using Orthogonal Partial Least Squares Discriminant Analyses (PLS-DA) (Rabbi et al., 2016). The principal component analysis revealed that samples collected at 3 and 6 h (0.5, 1, and 2 mg/ml) were more tightly grouped together (Figure 6A). In contrast, samples collected at the 24 h time point (0.5, 1, and 2 mg/ml) separated from the other time points and distributed in wider range. Next, we analyzed abundance of bacterial genera by one-way ANOVA, which revealed marked differences among nine genera. The analysis provided the trend of bacterial abundance on *Bacteriodetes, Afipia, Marivita, Turicibacter, Microcystis, Haemophilus, Sneathia, Lactobacillus*, and *Alkalibacter* in samples collected at the 24 h time point for all three concentrations (0.5, 1, and 2 mg/ml) were compared to samples collected at the 3 and 6 h time points (Figure 6B). Among 24 h samples, the control had a higher abundance of these bacterial genera than experimental groups for all three concentrations. More specifically, bacterial abundance in samples exposed to higher concentration of aloin (2 mg/ml) was affected the most. In contrast, the genus *Alkalibacter* was abundant in samples collected at 3 and 6 h time points for all three aloin concentrations. These results were consistent with bacterial growth curve data (for example *L. acidophilus*) generated in the present study. Subsequently, heat map was generated to define beta diversity (Figure 6C,D) that reveals overall genus abundance in the experimental groups, and abundance of top 50 genera, respectively.

### The Effect of Aloin on Bacterial Species Level as Assessed by 16s rRNA Sequencing

**FIGURE 7:**
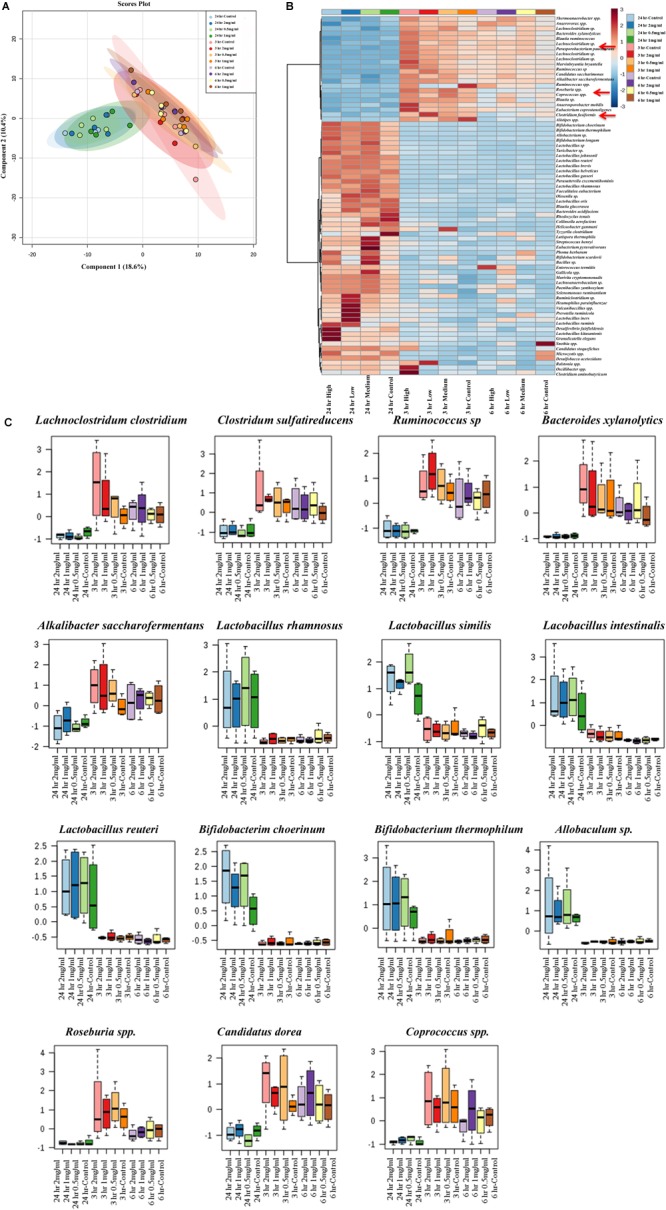
Bacterial community segregation at species level. **(A)** The PCA plot shows the clear segregation of bacterial community at species level at 24 h time point samples from the other two-time points. Samples collected 3 and 6 h time points are grouped tightly together. The PCA plots also reveal that diversification was more at species level than genera level. **(B)** Heat map shows the diversity among aloin treated experimental groups. The heat map shows the top 50 bacterial species that altered (either increased or decreased) due to aloin treatment. Here we have shown with red arrow mark of few bacterial species, which were decreased due to aloin treatment. These species are major contributors for butyrate production in the intestine. **(C)** These figures show that aloin exposure alters the bacterial community increased or decreased abundance at species level. Here we show representative examples for high as well as low abundance on bacterial species due to aloin treatment.

Next, analysis was focused on the bacterial species level to see whether there was any separation between experimental groups regarding specific bacterial species. The PLS-DA analysis displayed the differences in species level in samples that were exposed to aloin. The species separation was similar to genus level. Specifically, samples that were collected at the 24 h time point (all three concentrations) grouped together. In contrast, samples that were collected at 3 and 6 h grouped together and separated from 24 h time point samples (Figure 7A). We also observed a clear difference at the species level for the experimental groups.

To provide a clear picture on the effect of aloin at species level, the heat map was generated to provide beta diversity in the experimental groups (Figure 7B). The heat map provides a snap-shot of bacterial abundance at the species level and the difference within an experimental group and among the experimental groups. Here, we provided heat map data for the top 50 bacterial species that were affected by aloin treatment. The most significant representative bacterial species, in which aloin treatments had no adverse effect on *Lactobacillus rhamnosus, Lactobacillus similis, Lactobacillus Intestinalis*, and *L. reuteri* species, which showed abundance in the 24 h samples (Figure 7C) for all three aloin concentrations. Interestingly, aloin treatment increased the abundance of these bacterial species as compared to control. The 16s rRNA sequencing result is consistent with *L. acidophilus* growth curve data and live bacterial counts, where aloin either lacks microbicidal property or increases the bacterial growth. Similarly, *Bifidobacterium choerinum* and *B. thermophilum* also increased due to the aloin treatment at 24 h samples. In contrast, *Clostridium indolis, Clostridium sulfatireducens, Bacteroides xylanolytics*, and *Alkalibacter saccharofermentans* species decreased in abundance in samples collected at the 24 h time point (Figure 7C) for all three aloin concentrations. These bacterial species were abundant in samples collected at the 3 and 6 h time points for all three concentrations. We also observed that in live bacterial counts and bacterial growth curve, aloin had antimicrobial property at higher concentration, as well as during prolonged exposure (Figure 1C,F, 3A). The sequencing data very well support the *in vitro* experimental data of the present study.

### Effect of Aloin on Transepithelial Resistance

**FIGURE 8:**
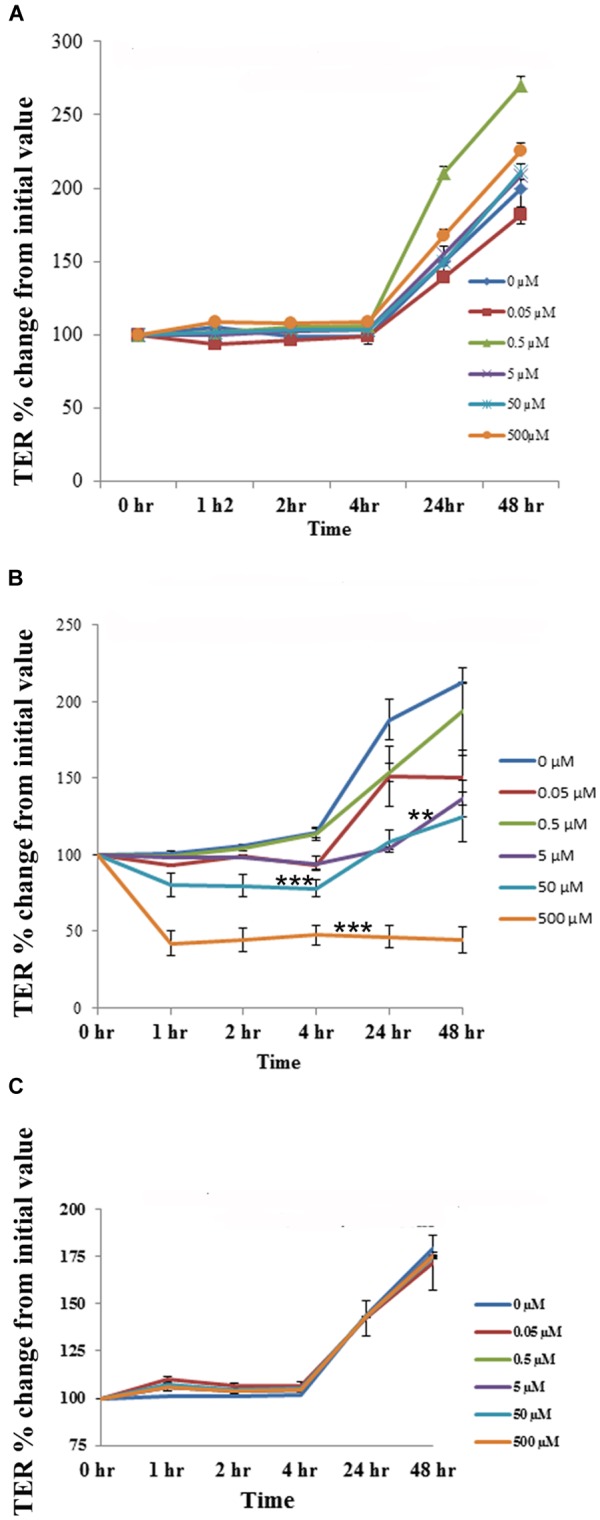
The effect of aloin on the permeability of human intestinal epithelial cells. T84 cells were grown in transwells till a TER value of 1,000 or above was reached. The TER value was recorded before and after incubation with five concentrations of aloin (0.05, 0.5, 5, 50, and 500 μM) at 0, 1, 2, 3, 24, and 48 h. The TER values recorded before adding aloin served as a baseline value. Data are presented here as % increase or decrease from the baseline readings. These data represent an average of seven independent experiments. The asterisk ^∗^ indicates statistical significance. **(A)** This graph shows the TER value for aloin dissolved in pH 7.2. **(B)** It shows the TER value for aloin dissolved in pH 4.9 citrate buffer and further diluted in cell culture media (antibiotic free media). **(C)** TER value for aloe-emodin. Asterisks indicates statistical significance (^∗^*p* < 0.05, ^∗∗^*p* < 0.01, ^∗∗∗^*p* < 0.001).

The cell cytotoxicity study revealed that aloin at higher concentrations induced cell death. Next, we tested whether low concentrations of aloin can have impact on the permeability of intestinal epithelial cells or not? In this experiment, we dissolved aloin at two different pH solutions (pH 4.9 and 7.2) and tested the intestinal barrier integrity to mimic the intestinal pH. When cells were exposed with aloin (0.05–500 μM) that was dissolved in pH 7.2 buffer, the transepithelial resistance increased similarly to the control indicating the barrier integrity remains intact (Figure 8A). In contrast, when cells were exposed to aloin at (0.05–500 μM) dissolved in pH 4.9 (then diluted into cell culture media so final pH was equal to culture media) the TER value decreased in a dose-dependent manner indicating compromised barrier integrity (Figure 8B). As aloin is metabolized by intestinal bacteria into aloe-emodin, we also tested the effect of aloe-emodin on intestinal cell integrity. Aloe-emodin was dissolved in ethanol and diluted into culture media (pH 7.2). The results revealed that aloe-emodin did not decrease the TER value indicating that intestinal barrier integrity remained intact (Figure 8C).

## Discussion

Pharmacological and phytochemical molecules extracted from Aloe Vera plants exhibit several beneficial properties (Briggs, 1995; Moore and Cowman, 2008). However, several studies also report adverse effects of aloin, for example, one human case study showed that oral consumption of 500 mg capsule of aloe extract for 4 weeks by a 56 years old woman resulted in acute hepatotoxicity; however, upon discontinuation of aloe capsules a rapid improvement from liver damage was noticed (Rabe et al., 2005). In another case study, a 24 years young adult consumed 500 mg of Aloe vera capsules for 3 weeks; liver biopsy from this individual revealed liver toxicity that was similar to drug induced liver toxicity as well as other clinical abnormalities (Kanat et al., 2006). Several other studies also provide evidence that human oral consumption of aloe vera resulted in the hepatotoxicity (Bottenberg et al., 2007; Yang et al., 2010; Lee et al., 2014).

Orally consumed aloe products reach the intestine where they interact with intestinal epithelial cells and inhabitant intestinal microbiota. Earlier studies have shown that aloin exhibits antimicrobial properties against pathogenic bacteria (Asamenew et al., 2011; Abeje et al., 2014); however, limited information is available on aloin antimicrobial properties on human intestinal commensal microbiota. The gut-liver axis with a focus on commensal microbiota, as well as, microbiota-derived factors has emerged as key player in the hepatic as well as intestinal diseases (Adolph et al., 2018). A balanced intestinal microbiota is essential for intestinal homeostasis and overall health. Thus, there is a need to address the knowledge gap between interactions of aloe derived products with effects on the intestine commensal microbiota. In this study, we addressed whether aloin has antimicrobial activity against intestinal microbiome. The experimental approaches used in this study were fivefold: (1) evaluate aloin antimicrobial property against selected intestinal commensal bacteria that are known to positively contribute to human health; (2) assess the effect of aloin on rat fecal samples that mimic the bacterial composition of human intestine under anaerobic condition to delineate the effect on intestinal commensal bacterial community; (3) to determine the effect of aloin on SCFAs production by intestinal commensal bacteria; (4) to assess if aloin changes the intestinal epithelial cell permeability and (5) calculate metabolism of aloin by intestinal commensal bacteria.

The human intestine is a residence for 35,000 bacterial species (Frank et al., 2007) that belong to 50 bacterial phyla (Schloss et al., 2005). Among them *Bacteroidetes* and *Firmicutes* are two major phyla that account for 90–95%, of the bacterial population (Gordon and Dubos, 1970). In addition, *Proteobacteria, Verrucomicrobia, Actinobacteria*, and other phyla also contribute to various functions in the intestine (Eckburg et al., 2005). In the present study, the 16S rRNA sequencing data indicates that *Bacteroidetes, Firmicutes*, and *Proteobacteria* contribute 90%, while *Verrucomicrobia* and *Actinobacteria* contribute for the remaining phyla found in rat fecal samples (Figure 3), which aligns with earlier reports (Fujio-Vejar et al., 2017). Commensal bacterial species are distributed differentially and colonized throughout the intestine. Rat bacterial phyla are more closely related to human than other murine species (Li et al., 2017). In this study, we tested aloin antibacterial effects against seven bacterial species (one pathogenic and six commensal bacteria) that belongs to five dominant bacterial phyla of human microbiota (Table 1). For the *in vitro* bacterial growth curve study, we used representative Gram-positive and Gram-negative commensal bacteria. The growth conditions tested of these bacterial species ranged from strict anaerobic to facultative anaerobic (Table 1).

**Table 1:** Aloin MIC value for intestinal commensal both Gram-positive and Gram-negative bacteria.

Bacterial species	Phylum	Gram +/-	Genus	Growth media	Conditions	MIC value
*Bacteroides thetaiotaomicron*	*Bacteroidetes*	-	*Bacteroides*	Modified chopped meat media	Anaerobic	2 mg/ml
*Enterococcus faecium*	*Firmicutes*	+	*Enterococcus*	Brain Heart infusion Broth	Facilitative	4 mg/ml
*Eubacterium* sp.	*Firmicutes*	+	*Eubacterium*	Base Cholesterol Medium	Anaerobic	2 mg/ml
*Lactobacillus acidophilus*	*Firmicutes*	+	*Lactobacillus*	MRS media	Facilitative	>8 mg no MIC
*Lactobacillus acidophilus*	*Firmicutes*	+	*Lactobacillus*	MRS media	Aerobic	1 mg/ml
*Akkermansia muciniphila*	*Verrucomicrobia*	-	*Akkermansia*	Brain Heart infusion Broth	Anaerobic	2 mg/ml
*Bifidobacterium longum*	*Actinobacteria*	+	*Bifidobacterium*	Brain Heart infusion Broth	Anaerobic	>8 mg no MIC
*Escherichia coli*	*Proteobacteria*	-	*Escherichia*	TSP media	Aerobic	1 mg/ml

Earlier, it was reported that aloin exhibits antibacterial activity toward Gram-negative bacteria (Asamenew et al., 2011; Mariappan and Shanthi, 2012), but is incapable of killing Gram-positive strain, such as *Bacillus pumilus* and *Bacillus subtilis* (Megeressa et al., 2015). In the present study, the *in vitro* bacterial growth curve shows that aloin also exhibits antimicrobial properties toward Gram-positive intestinal commensal bacteria, specifically *E. faecium* and *L. acidophilus.* However, aloin had limited antibacterial activity toward *B. longum*, another member of Gram-positive bacteria. Several intestinal bacteria are important for vitamin synthesis and degradation of undigested food materials (Wong et al., 2006). Earlier studies have shown that *Eubacterium* sp. is capable of degrading aloin into aloe-emodin and glucose, which is then utilized as an energy source for bacterial multiplication (Pogribna et al., 2008). Our results are consistent with an earlier report that *Eubacterium* spp. multiplication was directionally proportional to the concentration of aloin in the culture media (Che et al., 1991). Using non-animal model we also confirmed previous reports that aloin is metabolized into aloe-emodin or aloesin by intestinal bacteria (Che et al., 1991). These metabolites are structurally similar to anthraquinones and chromones, which exhibit antibacterial activity against Gram-positive and Gram-negative pathogenic bacteria (Budzisz et al., 2001; Cock, 2007). Aloin and its metabolites may function similar to anthraquinones or chromones toward intestinal commensal bacteria. The growth of *L. acidophilus, E. faecium*, and *B. thetaiotaomicron* were completely inhibited at 1 mg/ml concentration (Figure 1C,E,G), which indicates that aloin has similar levels of antibacterial activities to both Gram-positive and Gram-negative bacteria.

The molecular mechanism of antibacterial activity of aloin or its metabolites and anthraquinones is not well understood. However, several mechanisms have been put forth for the antimicrobial properties of aloin or its metabolites including the inhibition of membrane and respiration transport (Ubbink-Kok et al., 1986; Hamman, 2008). Aloin and its metabolites contain phenolic structures that are considered bioactive molecules, which may exhibit antimicrobial property against commensal bacteria. A recent study provided underlying molecular mechanisms of emodin’s (metabolites of aloin) antimicrobial property. Emodin, an anthraquinone, interacts with bacterial cell wall proteins, thereby increasing the permeability due to alteration in cell wall structural integrity. This causes an outflow of intracellular contents that may result in bacterial death (Li et al., 2016). Emodin causes bacteriostatic effects by inhibiting bacterial growth or multiplications at low concentrations (16 and 32 μg/ml) and causes the bactericidal effect at higher concentration (64 μg/ml) (Li et al., 2016). The growth kinetic results of the present study also provide evidence that aloin at low concentrations causes the bacteriostatic effect on *L. acidophilus* (*aerobic condition*), *E. faecium*, and *B. thetaiotaomicron* and bactericidal effect at higher concentrations. Nevertheless, the antibacterial property of aloin is species specific and is dependent on environmental factors such as aerobic and anaerobic growth conditions.

In the present study, the *L. acidophilus* grown in the presence of 1 mg/ml concentration of aloin experienced growth inhibition only under aerobic condition, whereas 2 mg/ml and above caused a bactericidal effect (Supplementary Figure S1). Interestingly, at low aloin concentrations, *L. acidophilus* appeared as a long chain of bacteria. This “chain-like” appearance could be correlated to bacterial aggregation caused by aloin or newly dividing bacteria unable to detach from each other in the presence of low concentrations of aloin (0.5 and 1 mg/ml). In contrast, bacteria exposed to a higher concentration of aloin lost their rod-shaped morphology and appeared as a “bristle-like” structure. We also examined the bactericidal mechanism of aloin by staining *L. acidophilus* with acridine orange and ethidium bromide after the completion of the growth curve experiment. Wells exposed to 0.5 mg/ml aloin revealed that all of *L. acidophilus* was stained green, whereas orange-stained bacteria were not observable. Acridine orange permeates intact cell membrane of rod shape bacteria, intercalates with DNA and fluoresces green in color, which is indicative of live *L. acidophilus*. Additionally, all bacteria retained rod shape morphology. This result suggests that low aloin concentration (0.5 mg/ml) is unable to induce killing mechanism or cytotoxicity. Wells exposed to 1 mg/ml showed 50% of bacteria with green and the remaining red in color. Bacteria red in color indicate that the ethidium bromide permeates bacteria that lost membrane integrity or in the process. In contrast, wells exposed with 2 mg/ml contained bacteria that were all red in color. Likely, they lost their cell wall structural integrity and formed bristle-like structures (Supplementary Figure S1) indicating that higher aloin concentration caused bactericidal effects. The possible mechanism of bactericidal effects that aloin may cause on bacterial membranes is very similar to emodin (Li et al., 2016).

Rat fecal samples that were exposed to various concentrations of aloin experienced changes in intestinal bacterial population. Fecal samples exposed to low aloin concentrations (0.5 and 1 mg/ml) underwent minor perturbation at the 3 and 6 h time points, whereas at 24 h bacterial colony forming units (CFU) were either increased or decreased depending upon the bacterial species. For example, under anaerobic growth conditions *Lactobacillus* CFU increased at 24 h time point, but not at the 3 or 6 h time point. These data are consistent with the *in vitro L. acidophilus* anaerobic growth curve result, in which we observed an initial 10 h lag phase following by an exponential growth (Figure 3C). The 16S rRNA sequencing data revealed the abundance of *Lactobacillus* species (Figure 6C) and supports the *in vitro* bacterial growth curve data and fecal live bacterial growth curve data. In the present study, we demonstrated that aloin exhibits antibacterial activity for some of the intestinal commensal bacteria, and the MIC value ranged from 1 mg to 4 mg/ml. Concentrated aloe gel or powdered form of leaf extracts are available in the market as a capsule containing 100–500 mg dose. Whereas, liquid consumption results in exposure to approximately 14.4 g whole leaf extract. In these consumer use products, aloin, levels range from 0.1 to 6.6% of leaf dry weight (Groom and Reynolds, 1987); thus indicating presence of aloin in a concentration range between 0.1 and 35 mg. However, to our knowledge, data is lacking on what concentration levels reach the GIT.

Next, we analyzed the effect of aloin on SCFAs production by intestinal commensal bacteria, which is an essential energy source for intestinal epithelial cells and gut homeostasis. The HPLC data provides evidence that microbial derived butyrate production had decreased at the 24 h time point in fecal samples treated with aloin. The sequencing data further confirms a decrease abundance of butyrate producing bacterial species (*Clostridium* spp., *Roseburia* spp., *Coprococcus* spp., and *Eubacterium* spp.) at 24 h time point, (Figure 6C). The depletion of bacterial species occurs more readily in aloin treated samples than control samples. A recent study suggests that streptomycin exposure in mice reduced butyrate producing *Clostridia* in the intestine within a day, which resulted in decreasing butyrate concentration by fourfold in cecum and facilitated the expansion of aerobic pathogenic *S.* Typhimurium (Rivera-Chavez et al., 2016). In the intestine *Clostridia* spp. contributes a substantial amount of butyrate (Louis and Flint, 2009; Vital et al., 2014), which is consumed by colonocytes to create a hypoxic environment (Kelly et al., 2015) in the intestine by converting butyrate into CO_2_ (Donohoe et al., 2012). Change in the intestinal bacterial community structure has been strongly correlated to colonic disease and irritable bowel syndrome (IBS) primarily due to a decrease in the production of butyrate (Sokol et al., 2009; Eeckhaut et al., 2013). In ulcerative colitis patients with a reduced butyrate production, the depletion of *Clostridium coccoides* and *Clostridium leptum* was a probable contributing factor for etiology (Kumari et al., 2013). In addition, intestinal microbial derived butyrate increases the mitochondrial dependent oxygen consummation and inhibits the proinflammatory mediator expression through histone deaceytlase (Davie, 2003; Furusawa et al., 2013; Zheng et al., 2017). These studies suggest the importance of intestinal microbial derived butyrate on intestinal homeostasis. In the present study, decreased butyrate production could be correlated to the decreased abundance of butyrate-producing bacterial species. We show that the high concentration and long-term exposure of aloin decreased the butyrate production in rat fecal samples. The decreased butyrate production by intestinal commensal bacteria can be correlated to development of intestinal abnormalities reported in animal studies (Boudreau et al., 2013, 2017). The sequencing data complements SCFAs production and provides evidence for the antibacterial property of aloin on SCFA producing commensal bacteria. Furthermore, butyrate provides an energy source for intestinal epithelial cells and promotes epithelial barrier formation by decreasing the expression of pore forming claudin-2 genes (Zheng et al., 2017) and suppressing the colonic inflammation and carcinogenesis through the activation of GPA109a receptor (Singh et al., 2014).

Intestinal transepithelial resistance experiments revealed that aloin decreases the permeability barrier function only at high concentrations. Aloin dissolved in pH 4.9 decreased TER value indicating that barrier function is compromised within 2 h of exposure. It has been shown that aloin is more stable in acidic pH (pH 2.0) for a longer period compared to in basic pH (pH 8.0) (Ding et al., 2014). Aloin dissolved at pH 4.9 may be more stable than at neutral pH 7.2. The compromised barrier function may be associated with the stability of aloin at an acidic pH 4.9. Aloe-emodin treated cells maintained the intestinal barrier integrity. The focus was to evaluate the effect of aloin on intestinal epithelial cells; hence, we have not tested its properties by dissolving aloe-emodin in acidic pH 4.9. Aloin cytotoxicity was also assessed using polarized intestinal epithelial cells. Polarized intestinal epithelial cells were exposed to the same concentrations that were used in intestinal commensal bacteria. To differentiate live and dead cells, acridine orange and ethidium bromide were added as described earlier (Williams et al., 2015). The microscopic examination reveals that 2 mg/ml (4.78 mM concentration) aloin exposed cells were stained 50% green in color and remaining cells red color. In the TER experiment, 500 μM aloin compromised the barrier function. Thus, a greater concentration of aloin (i.e., 2 mg/ml or 4.87 mM) will have a greater detrimental effect on epithelial cells and may result in cytotoxicity. Earlier, it has been shown that Jarkat T-lymphocytes exposed to aloin caused several abnormalities including altered cellular morphology, cell cycle arrest, loss of membrane integrity, and induced cytotoxicity by apoptotic mechanism (Buenz, 2008). The present study also demonstrated that aloin induced cytotoxicity in intestinal epithelial cells required at least two times higher concentration than the effect observed in Jarkat T-lymphocytes. The possible mechanism may involve intestinal epithelial cells secreting mucin, which could serve as a protective layer and prevent immediate interaction with epithelial cells. In contrast, T-lymphocytes lack a mucus layer, hence, aloin may interact with T-cells more quickly and require a smaller aloin concentration to induce cytotoxicity.

*Bifidobacterium* contributes several beneficial functions to the intestine. To date, 48 species have been recognized in this genus. Genome sequence analysis reveals presence of genes that can encode cell surface macromolecule proteins, which possibly play a role in bacterial attachment and colonization in the intestine (Dethlefsen et al., 2008). *Bifidobacterium* constantly encounters oxidative stress, and exposure to free radicals, various intestinal enzymes, and bile acids that can have a detrimental effect on bacterial survival and intestinal mucosa attachment. In addition, consumption of antibiotics and xenobiotic compounds has a negative impact on colonization and survival of *Bifidobacterium*. The present study provides evidence for the antimicrobial property of aloin to commensal bacteria that were cultured under anaerobic conditions. Specifically, pure *in vitro* cultured *Bifidobacterium* showed decreased bacterial growth or CFU in a dose- dependent manner. Aloin may bind to bacterial cell wall and cause the bactericidal effect by altering the bacterial membrane structural integrity; that could result in dysbiosis state. Due to this dysbiosis intestinal mucosa could become more prone to aloin mediated injury. This could probably be also a reason for the development of intestinal lesions and goblet cell hyperplasia in ascending colon in rats exposed to aloe whole leaf extracts or purified aloin compound in earlier animal studies (Boudreau et al., 2013, 2017).

In animal studies, the concentration of aloin available to interact with intestinal microbiota is very difficult to determine, as the oral exposure is usually through drinking water, where the aloin consumption and availability in the intestine to interact with intestinal microbiome could vary in each animal. Moreover, information is required for the SCFA metabolism to correlate active microbiome and the healthy gut. The non-animal models used in the current study (specific bacteria-aloin interaction and fecal slurry-aloin interaction model) address these knowledge gaps. Furthermore, using *in vitro* cultured human intestinal epithelial cells, we also showed a direct effect of aloin on the gut permeability. This study provides clear evidence that due to aloin (1 mg/ml and above) treatment several bacterial species show a low abundance that are involved in the butyrate production. Furthermore, we show that the butyrate production was decreased due to aloin treatment. Thus, the microbial 16s sequencing data complements the biochemical data (butyrate production) during the aloin treatment. Overall our study shows that aloin possibly may cause toxic effect; however, it depends upon aloin concentration.

## Conclusion

The present study provides evidence that aloin exhibits antibacterial properties toward intestinal commensal bacteria, depending upon the growth conditions and concentration of aloin. In addition, aloin exposure decreased butyrate production by decreasing the abundance of butyrate producing bacterial species. The quantification of butyrate by HPLC analytical method confirmed decreased level of aloin exposure for 24 h. The sequencing data further supports microbiological and biochemical data observed in the present study. Transepithelial resistance results provide evidence that aloin dissolved at pH 4.9 compromised the intestinal barrier function in a dose -dependent manner.

## Author Contributions

KG, CC, and SK conceived and designed the experiments. KG, PK, and SK performed the experiments. KG and SK analyzed the data and contributed reagents, materials, and analysis tools. KG, CC, and SK wrote, reviewed, and approved the manuscript.

## Disclaimer

The findings and conclusions presented in this manuscript are those of the authors and do not necessarily represent the views of the United States Food and Drug Administration.

## Conflict of Interest Statement

The authors declare that the research was conducted in the absence of any commercial or financial relationships that could be construed as a potential conflict of interest.

## Supplementary Material

FIGURE S1 Effect of aloin on the morphology and survival of *Lactobacillus acidophilus* after incubation aloin treatment. The figures shown are the picture taken from the wells treated without aloin **(A)** or with aloin [**B–D** (0.5, 1, and 2 mg/ml)]. Control wells had individual rod shape bacteria, in contrast, wells treated with 0.5 mg and 1 mg/ml had long chain like appearance due to bacterial attachment or unseparated multiplying bacteria. At higher concentration of aloin treatment the bacteria lost its morphology and cellular content and appeared as a bristle like structure. The bottom panels **(E–G)** was stained with acridine orange and ethidium bromide for 15 min before images were captured. Bacteria stained green in color were live, whereas dead bacteria stained orange. Wells with 0.5 mg/ml of aloin showed all bacteria stained green, indicating the live bacteria; whereas, 2 mg/ml treated wells had bristle like appearance and stained orange indicating dead bacteria with change in morphology.
