# Supplementary figures and images for: Dose-Dependent Effects of Aloin on the Intestinal Bacterial Community Structure, Short Chain Fatty Acids Metabolism and Intestinal Epithelial Cell Permeability
Source: Front Microbiol. 2019 Mar 26;10:474. doi: 10.3389/fmicb.2019.00474 (PMC6443721; doi:10.3389/fmicb.2019.00474)

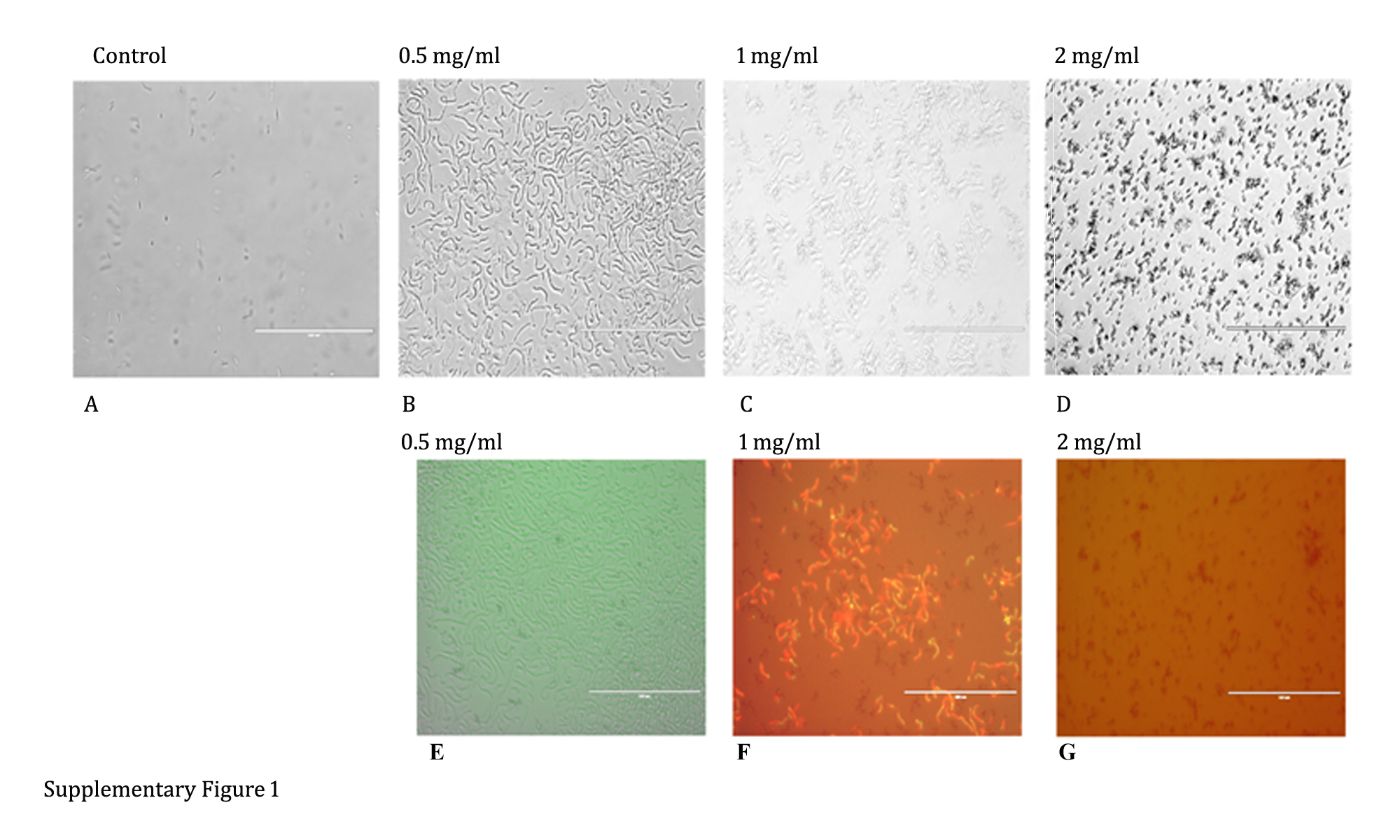

Supplement: FIGURE S1 — Effect of aloin on the morphology and survival of Lactobacillus acidophilus after incubation aloin treatment. The figures shown are the picture taken from the wells treated without aloin (A) or with aloin [B–D (0.5, 1, and 2 mg/ml)]. Control wells had individual rod shape bacteria, in contrast, wells treated with 0.5 mg and 1 mg/ml had long chain like appearance due to bacterial attachment or unseparated multiplying bacteria. At higher concentration of aloin treatment the bacteria lost its morphology and cellular content and appeared as a bristle like structure. The bottom panels (E–G) was stained with acridine orange and ethidium bromide for 15 min before images were captured. Bacteria stained green in color were live, whereas dead bacteria stained orange. Wells with 0.5 mg/ml of aloin showed all bacteria stained green, indicating the live bacteria; whereas, 2 mg/ml treated wells had bristle like appearance and stained orange indicating dead bacteria with change in morphology. [file Image_1.TIF]
